# GAIL: An interactive webserver for inference and dynamic visualization of gene-gene associations based on gene ontology guided mining of biomedical literature

**DOI:** 10.1371/journal.pone.0219195

**Published:** 2019-07-01

**Authors:** Daniel Couch, Zhenning Yu, Jin Hyun Nam, Carter Allen, Paula S. Ramos, Willian A. da Silveira, Kelly J. Hunt, Edward S. Hazard, Gary Hardiman, Andrew Lawson, Dongjun Chung

**Affiliations:** 1 Department of Public Health Sciences, Medical University of South Carolina, Charleston, SC, United States of America; 2 Department of Medicine, Medical University of South Carolina, Charleston, SC, United States of America; 3 Department of Pathology and Laboratory Medicine, Medical University of South Carolina, Charleston, SC, United States of America; 4 Center for Genomic Medicine, Medical University of South Carolina, Charleston, SC, United States of America; University of the Sunshine Coast, AUSTRALIA

## Abstract

In systems biology, inference of functional associations among genes is compelling because the construction of functional association networks facilitates biomarker discovery. Specifically, such gene associations in human can help identify putative biomarkers that can be used as diagnostic tools in treating patients. Although biomedical literature is considered a valuable data source for this task, currently only a limited number of webservers are available for mining gene-gene associations from the vast amount of biomedical literature using text mining techniques. Moreover, these webservers often have limited coverage of biomedical literature and also lack efficient and user-friendly tools to interpret and visualize mined relationships among genes. To address these limitations, we developed GAIL (Gene-gene Association Inference based on biomedical Literature), an interactive webserver that infers human gene-gene associations from Gene Ontology (GO) guided biomedical literature mining and provides dynamic visualization of the resulting association networks and various gene set enrichment analysis tools. We evaluate the utility and performance of GAIL with applications to gene signatures associated with systemic lupus erythematosus and breast cancer. Results show that GAIL allows effective interrogation and visualization of gene-gene networks and their subnetworks, which facilitates biological understanding of gene-gene associations. GAIL is available at http://chunglab.io/GAIL/.

## Introduction

Systems biology is currently believed to be dictated by ‘functional modules,’ groups of gene products that are functionally similar and together contribute to some high-level function [1]. Due to the importance of functional modules, one major goal in systems biology is to construct genome-wide functional association networks among genes [2], especially for humans. For example, network analysis has led to the discovery of network-based biomarkers [3, 4]. Since genes involved in a disease tend to be associated with one another, forming so-called ‘disease modules,’ understanding these modules is imperative to precision medicine in the future [5, 6].

In a functional association network, each gene is represented as a node and the functional similarity between any two genes is represented as a weighted edge, where the edge weight measures the gene-gene functional similarity. Often, a threshold value for the functional similarity measure is specified when displaying an association network. In this case, an edge is present only if the functional similarity between two genes exceeds the threshold. The major data sources to infer gene-gene association networks are experimental data such as gene co-expression and quantitative mass spectrometry on proteins, two hybrid screening [7, 8] and co-immunoprecipitation data [9, 10], as well as databases indicating biological pathway co-membership between genes [11–13]. Another valuable data source to infer gene-gene association is the biomedical literature, including abstracts, full-length texts, and text annotations [14]. One of the most popular data sources for biomedical literature is the PubMed database (https://www.ncbi.nlm.nih.gov/pubmed), which contains more than 27 million articles.

Compared to experimental data, biomedical text data potentially provides more comprehensive information about gene-gene associations because it is not restricted to a certain experimental approach and reflects a long history of biomedical research. However, extracting information from biomedical text data is usually more challenging than analyzing experimental data because biomedical texts are less structured and the information is buried as fragments in free texts. Hence, in order to discover interesting information from the enormous amount of biomedical text, advanced techniques such as text mining need to be used [15]. One popular text mining approach is co-occurrence based mining, which searches for the appearance of two biomedical terms in excerpts of literature such as abstracts [16].

Although many webservers use PubMed articles as a raw data source to discover various biological relationships, a relatively small number of webservers are dedicated to text mining to infer gene-gene relationships and actively maintained. The Literome project is one such webserver, providing an automated curation system for directed genic interactions [17]. Specifically, given two genes, Literome identifies PubMed articles that suggest an interaction between the genes as well as potential gene mediators, and provides supporting excerpts from the articles used as evidence. However, Literome is essentially designed for deep investigation of a small set of genes and identification of relevant literature, but not for global inference of gene-gene relationships based on a large set of biomedical literature. PubTator [18, 19] is similar to Literome in that it assists the curation of biomedical relationships, but has the same limitation of not providing a global gene-gene network. Another relevant tool is GeneDive, which allows users to interactively visualize disease-gene-drug relations inferred from the DeepDive text mining method and provides basic network analysis features [20, 21]. However, the DeepDive method only looks at gene co-occurrence in literature articles, yet genes with similar functions do not necessarily appear together. This can potentially limit comprehensiveness of the text mining results. In addition, while GeneDive does provide some basic network visualization and analysis tools, interpretation of the results generated by GeneDive is still not straightforward for users. This is because the tool displays a large gene-gene network without providing any tools to investigate functional description of the genes shown. Overall, it is apparent that a gap exists in the current set of tools available for inferring gene-gene relationships using biomedical text mining.

In this paper, we present GAIL (**G**ene-gene **A**ssociation **I**nference based on biomedical **L**iterature), an interactive and user-friendly webserver to infer and visualize human gene-gene associations. GAIL provides several features that are currently not provided by other biomedical literature mining webservers. First, we extended our text mining results by utilizing gene ontology (GO) terms because GO terms can improve gene-gene association inference compared to text mining approaches that are based only on co-occurrence of gene names [22]. The use of GO terms in constructing gene-gene relations also enables GO-based network analysis, making the results more interpretable from a systems biology perspective. Second, GAIL provides comprehensive network visualization and inference tools, including a ‘community detection’ feature, in which the network topology is analyzed to give the user the relevant communities (i.e., clusters) of genes within the whole network. GO analysis can be further implemented for these communities within the GAIL framework, which facilitates biological interpretation of the identified communities. Finally, users can download multiple types of lower-level data, which can be used for various downstream analyses. For example, the gene-gene association network data can be downloaded and used as prior information in fitting Bayesian models for gene regulatory network inference [23]. Likewise, users can also download a gene-GO association matrix and utilize it for various tasks such as classification and clustering analyses [24]. GAIL is available at http://chunglab.io/GAIL/.

## Results

### GAIL web interface and analysis workflow

**Fig 1:**
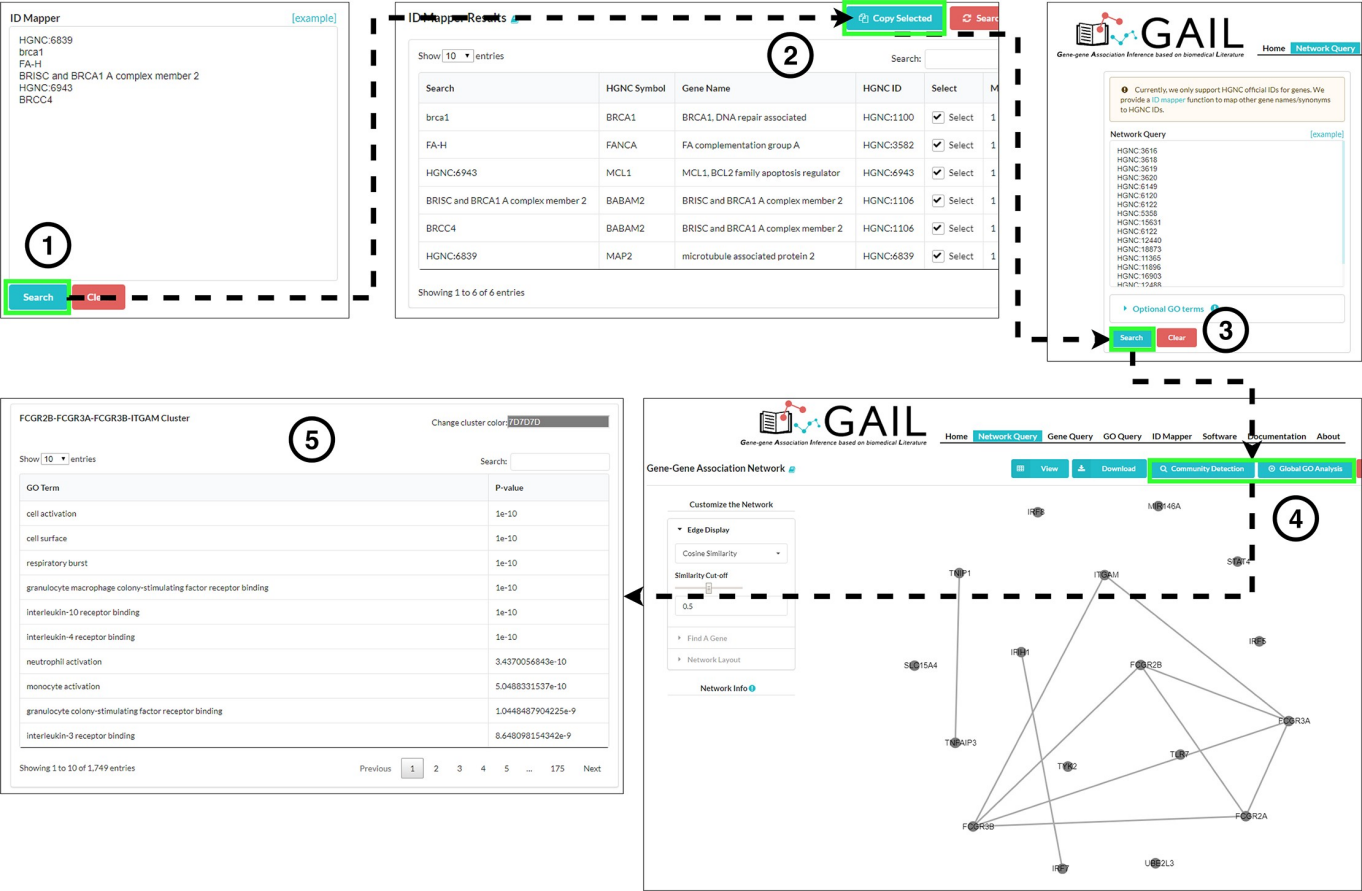
The analysis workflow of GAIL gene-gene association network query. Since the current network query only supports HGNC IDs, users can first map other gene symbols or synonyms to HGNC IDs using the ID Mapper (Step 1) and copy them to clipboard (Step 2). Next, users input these HGNC IDs and query the association network (Step 3). The gene-gene association network will display as in Step 4. Users can perform various types of network analysis and detect subgroups in the gene-gene association network (Step 5).

GAIL currently offers four queries: *Network Query*, *Gene Query*, *GO Query*, and *Gene-GO Query*. The *Network Query* page is the main functionality of GAIL, and it allows users to input a list of genes and implements interactive visualization and inference of association network for the given genes. **Fig 1** shows the workflow of Network Query. The *Network Query* page provides various options to improve visualization and implement inferences on gene-gene associations. First, users can change a cutoff to modify the network, showing only the edges of which similarity measures are greater or equal to the cut-off value. Second, users can change the network layout by changing the “Node Distance” value. Essentially, the greater the value is, the more dispersed the genes will be, while the smaller the value is, the denser the genes will be. This allows the user to visually check the gene clustering structure. Third, clicking the “Community Detection” button allows the user to implement a more rigorous approach to identify gene clusters. After running the community detection, the user can check members of each community (gene cluster). The user can also easily investigate biological functions related to each community by clicking the “GO Analysis” button. Fourth, this *Network Query* page provides various functions for the user to interrogate genes more closely. Typing the gene name in the panel “Find A Gene” allows the user to zoom in the network by putting the gene of interest in the center. If the user clicks a node, information about the gene with the link to GeneCards [25] will be provided, along with the top GO terms associated with the gene. Similarly, selecting an edge between genes will provide the user with information about the relationship between these two genes. Here, the user can also compare the GO term association patterns between two genes by clicking the “Check Shared GOs” button. Finally, users can download the table of cosine similarity values for downstream network analyses.

In the *Gene Query* page, users can query a list of genes. Then, the resulting page has a sidebar containing a list of the gene names and selecting one of these genes will show the GO terms associated with the gene. Here the user can find original *p*-values and Bonferroni-adjusted *p*-values for the association of each GO term with the gene based on the hypergeometric test. By default, the *p*-values will be displayed in order of significance, with the most significantly associated GO terms at the head of the table, so that the user can check the most important GO terms easily. The user can also filter the GO terms by name and download the table while he/she can check more information about the gene itself using a link to GeneCards [25]. On the other hand, in the *GO Query* page, users can query a list of GO terms and then investigate the genes associated with these GO terms. As in the case of the *Gene Query* page, this page will provide original *p*-values and Bonferroni-adjusted *p*-values for the association of each gene associated with the GO term, along with links to the corresponding GO term page on the Gene Ontology Consortium website [26].

The *Gene-GO Query* page takes a list of genes and GO terms as input and returns a table summarizing the hypergeometric test *p*-value computed between each gene and GO term. The data is downloadable either in the form of a table or as a matrix of *p*-values between genes and GO terms, allowing it to be used for downstream analyses. Alternatively, the user can also query all the GO terms. If the user is only interested in a limited number of the ‘most significant’ GO terms associated with the genes of interest, they can specify this using ‘# of GO terms’. Then, GAIL will provide the specified number of GO terms with the smallest average *p*-values for the input genes. In the current implementation of GAIL, we restricted the queried gene terms to be the HUGO Gene Nomenclature Committee (HGNC) IDs to improve speed of database retrieval. GAIL provides the *ID Mapper* functionality, to assist users who are more familiar with other gene synonyms. The ID mapper takes gene synonyms as inputs and returns corresponding HGNC IDs for these genes. Finally, users can find comprehensive documentation for all the utilities of GAIL on the webserver as well.

### Exploratory analysis of GAIL cosine similarity measures

**Fig 2:**
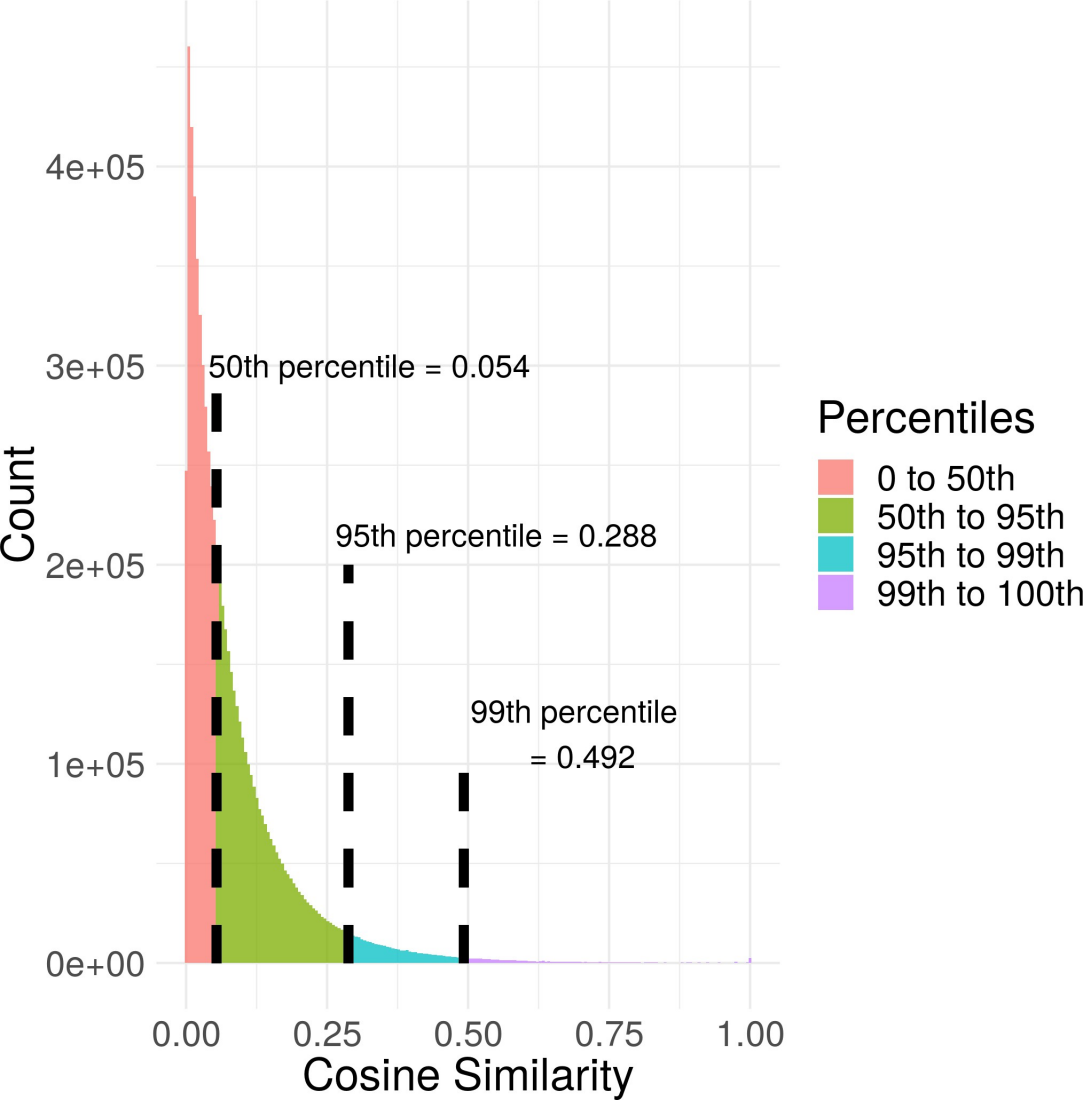
Distribution of cosine similarity values in the GAIL database. The cosine similarity values corresponding to 50^th^, 95^th^, and 99^th^ percentiles are also provided.

To guide decisions on cosine similarity cutoff points used for constructing gene networks, we investigated the distribution of cosine similarities in the GAIL database. We checked the empirical distribution of cosine similarities corresponding to each of the 7,069,574 unique pairs of genes in the database and found percentiles of interest (**Fig 2**). As expected, we observed a highly skewed distribution of cosine similarity measures. The 50^th^ percentile corresponds to a cosine similarity of 0.054, the 95^th^ percentile corresponds to a cosine similarity of 0.288, and the 99^th^ percentile corresponds to a cosine similarity of 0.492. Using these percentiles, users can control the confidence level of their visualization and inference. For example, if the users set the cosine similarity cutoff to 0.492, then the visualization and inference will be made based on the top 1% most confident edges.

### Comparison to co-occurrence approaches

In GAIL, we infer relationships among genes indirectly using the information available from GO terms. Our expectation is that this will result in a richer gene network compared to the approaches based on co-occurrence of gene names. We tested this hypothesis using the PAM50 signature genes that characterize breast cancer subtypes [27] (**S1 Table** in the Supplementary Information, and as detailed in Materials and Methods). Specifically, we constructed a network based only on co-occurrences of genes in an abstract (i.e., without utilizing GO terms) and compared the resulting network to the network produced by GAIL that utilizes GO terms. To ensure comparability between the two networks, we computed cutoff value (which determine edges) for each of GAIL and the co-occurrence-based approach separately using corresponding cosine similarity distribution. We considered cutoff values over a range of percentiles from 0.9 to 1.0, which are usually used in practice. Then, we compared the number of edges observed in the co-occurrence-based network with the number of edges observed in the network produced by GAIL. As expected, the network produced by GAIL included more edges than the co-occurrence-based network over the entire grid of cosine similarity percentiles between 0.9 and 1.0 (**S1 Fig** in the Supplementary Information). This result shows GAIL’s ability to produce richer gene-gene networks compared to traditional co-occurrence-based approaches.

### Application to the gene signatures associated with SLE

**Fig 3:**
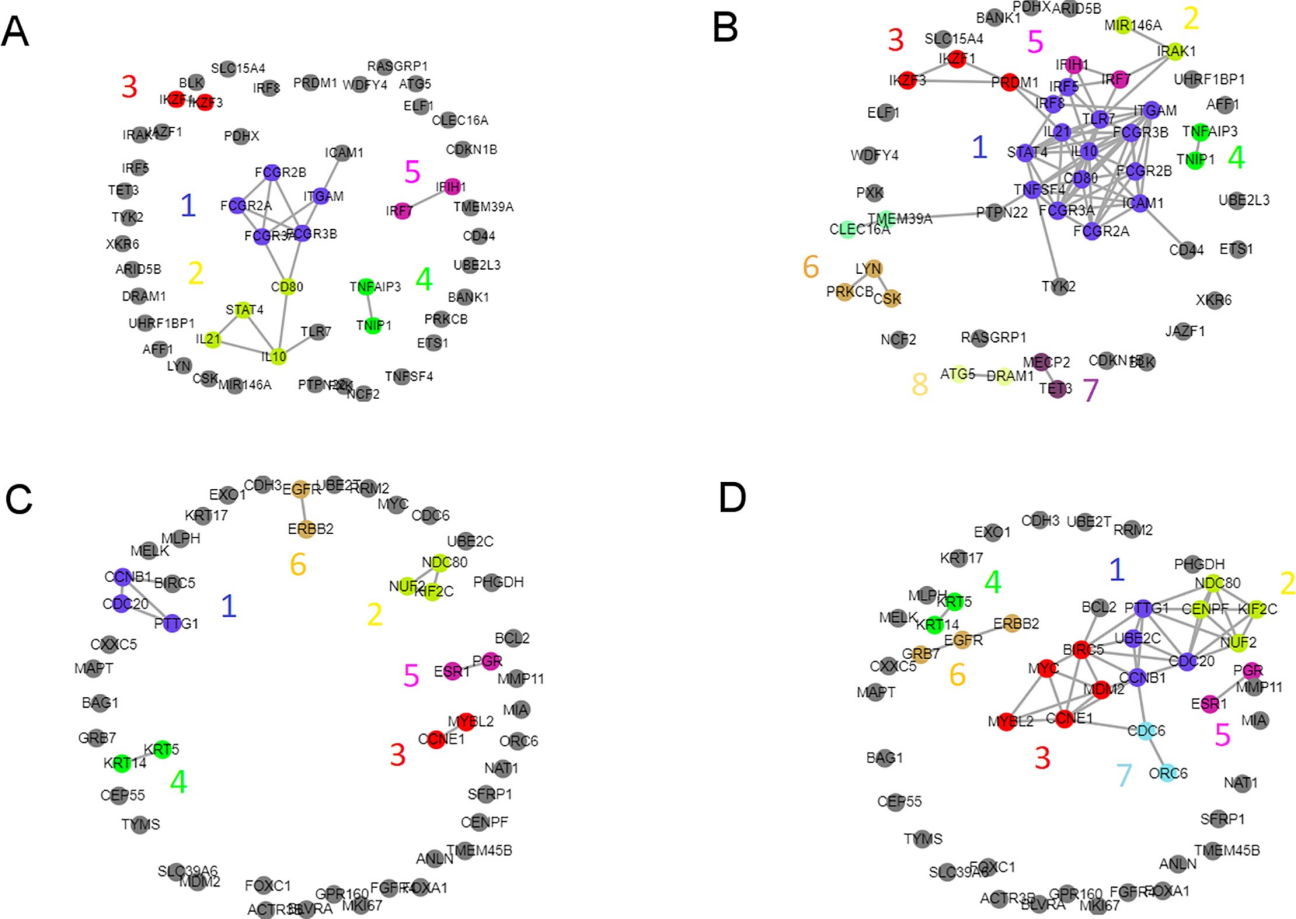
The gene-gene association networks produced by GAIL. The networks produced by GAIL for the gene signatures associated with SLE (A: high confidence, B: moderate confidence) and breast cancer (C: high confidence, D: moderate confidence). Colors indicate clusters identified using the ‘Community Detection’ function in GAIL. Here, high and moderate confidences mean networks constructed with the top 1% and 5% most confident edges, respectively.

The utility and performance of GAIL was evaluated using a published list of 51 gene signatures associated with systemic lupus erythematosus (SLE, or lupus) [28] (**S2 Table** in the Supplementary Information). Using a cosine similarity cut-off > 0.492 to display a network with the top 1% edges (**Fig 3A**: "high confidence level") yielded several networks. With this confidence level, most of the genes (39/55) were not associated with other genes, including the 12 genes that were not placed in any disease pathway [28]. Using the *Community Detection* function, five network clusters were unveiled. Interestingly, all the genes in the “Immune complex clearance” pathway reported in Deng and Tsao [28] (detailed in Materials and Methods) form the largest cluster, namely Cluster 1. Cluster 1 (*FCGR2A-FCGR2B-FCGR3A-FCGR3B-ITGAM*) is characterized by the genes’ roles in cell activation, as shown by their significant association with *cell activation* and *cytokine receptor binding (e*.*g*., *interleukin receptor binding* and *granulocyte macrophage colony-stimulating factor (GM-CSF) receptor binding)* GO terms. The reported “B and T cell signaling” genes are split into two clusters (Clusters 2 and 3). Cluster 2 (*CD80-IL10-IL21-STAT4*) is defined by the genes’ involvement in cytokine signaling, as demonstrated by their significant association with *cytokine production* and *interleukin receptor binding* GO terms. Cluster 3 (*IKZF1-IKZF3*) is marked by the genes’ roles in *lymphocyte (B and T cell) differentiation*. From the reported “NF-kB signaling” pathway, the genes coding for the interacting *TNFAIP3* and *TNIP1* molecules form Cluster 4, marked by their enrichment in *interleukin* and *NF-kB* GO terms. Finally, two of the reported “Type I interferon signaling” genes form Cluster 5 (*IFIH1-IRF7*), which is enriched with *interferon* and *viral response* GO terms.

Relaxing to a cosine similarity cut-off > 0.288 to display networks with the top 5% edges (**Fig 3B:** "moderate confidence level") joined the *FCGR2A-FCGR2B-FCGR3A-FCGR3B-ITGAM* and the *CD80-IL10-IL21-STAT4* cluster, further adding *IRF5*, IRF8, *TLR7*, *TNFSF4* and *ICAM1* (Cluster 1). This large cluster is marked by the association of *cytokine* GO term. This cluster provides support for the overlap between pathways because these genes were originally placed in each of the “Immune complex clearance” pathway (*FCGR2A*, *FCGR2B*, *FCGR3A*, *FCGR3B* and *ITGAM*), “Lymphocyte signaling” pathway (*IL10*, *IL21*, *CD80* and *TNFSF4*), and “Type I interferon” pathway (*STAT4*, *IRF5*, *IRF8* and *TLR7*). Such overlap was also observed in new Cluster 2 (*IRAK1*-*MIR146A*), of which genes were originally placed in the “NF-kB” and “Type I interferon” pathways, but together show a significant association of *innate immune response*, *interleukin-6*, *toll-like receptor*, and *NF-kB* GO terms. Cluster 3 (the previous *IKZF1-IKZF3* cluster, which was expanded by including *PRDM1*) remains marked by the significant association of *lymphocyte differentiation* GO term. The reported “B and T cell signaling” pathway formed a distinct Cluster 6 (*CSK*-*LYN*-*PRKCB*) defined by *signal transduction*, *kinase activity* and *phosphorylation* GO terms. These results underscore the utility of GAIL to unveil gene relationships.

### Application to the gene signatures associated with breast cancer

We next considered the PAM50 signature genes which characterize breast cancer subtypes [27] (**S1 Table** in the Supplementary Information). Using the cosine similarity cut-off > 0.492 and the *Community Detection* function applied to the PAM 50 signature genes (**Fig 3C**), six gene clusters were identified while 36/50 genes were not linked to any other genes. Cluster 1 (*CDC20*, *CCNB1*, *PTTG1*) is associated with *anaphase-promoting complex* GO term. Based on gene expression profiles, these genes are considered to be the same gene cluster that differentiates the molecular breast cancer subtypes and these genes are principally expressed in the Basal-like subtype but also in some Luminal A and some HER2-enriched patients [29]. Cluster 2 (*KIF2C*, *NDC80*, *NUF2*) is also in agreement with the molecular subtypes and has the expression pattern similar to Cluster 1 in breast cancer patients [29]. These genes are associated with *attachment of spindle microtubules to kinetochore*, a mechanism involved in preventing premature anaphase initiation [30]. Cluster 3 (*CCNE1* and *MYBL2*) has the same pattern of expression as Clusters 1 and 2 in breast cancer patients [31] and is associated with *regulation of cell cycle phase transition* GO term. Interestingly, all of these three clusters are related to the cell cycle but correspond to different cell cycle stages. Cluster 4 (*KRT5* and *KRT14*) is associated with *cytoskeleton intermediate filaments* and *keratinocyte differentiation* GO terms. These two genes show similar gene expression patterns among patients [31] and are expressed principally in Luminal A and Basal-like subtypes. They correspond to the more differentiated and the least differentiated subtypes, respectively [31, 32], which could indicate context-dependent roles of the genes in this cluster. Cluster 5 (*ESR1* and *PGR*) is associated with *estrogen receptor activity* and *mammary gland development* GO terms, and these genes are expressed in patients of the Luminal A and Luminal B subtypes [31]. Cluster 6 (*EGFR* and *ERBB2*) is the only group of which genes are expressed for different molecular subtypes: *EGFR* is expressed in Basal-like tumors and *ERBB2* in HER2-enriched tumors [31]. Nonetheless, these genes result in proteins that dimerize at the cell membrane while both of them belong to the human epidermal growth factor receptor (HER/EGFR/ERBB) family [33]. Genes in this cluster are associated with the *ERBB signaling pathway* and *epithelial to mesenchymal transition* GO terms.

When the cosine similarity cut-off was relaxed to 0.288 (**Fig 3D**), each of Clusters 1, 2, 3 and 6 was connected to a larger number of genes. Specifically, *UBE2C* was added to Cluster 1 and *CENPF* was added to Cluster 2, while Cluster 3 had new connections to *BIRC5*, *MDM2* and *MYC*. *BIRC5* shares the same gene expression pattern with the other members in Cluster 3 (*CCNE1* and *MYBL2*). On the other hand, *MDM2* is not expressed in Basal-like but in some Luminal A and Luminal B tumors while *MYC* is expressed only in Basal-like and some Luminal A tumors. *MYC* is one of the most studied oncogenes and its expression is linked to a high rate of cell proliferation [34]. This altogether might indicate that Cluster 3 as a whole regulates the rate of cell division and it might be of interest to investigate its members as targets for chemotherapy combination to avoid drug resistant tumors. Cluster 6 is now connected to *GRB7*, which is expressed on the HER2-enriched tumors [31] and recruited by *ERBB2* as a signaling protein [35]. For this new cutoff of 0.288, we also have one additional cluster, Cluster 7, which contains *CDC6* and *ORC6*. These genes are associated with *DNA replication*, another important step in the cell cycle, and they are principally expressed in Basal-like and Her2-enriched tumors.

Overall, GAIL was able to identify two important themes for cancer progression and aggressiveness. Specifically, Clusters 1, 2, 3 and 7 are involved in different steps of the cell cycle while Clusters 4, 5 and 6 are related to different aspects of cell differentiation in mammary gland. Analysis of the breast cancer signature genes using GAIL was not only able to reproduce the findings observed in patients [31], but was also able to elucidate different aspects of the breast cancer biology, which can lead to improved strategies for new personalized treatments.

### Downstream analysis using the downloaded data

One benefit of GAIL is that it allows users to download the lower-level data of hypergeometric test *p*-values between genes and GO terms. In this section, we show some potential utilization of this lower-level data. For this application, we first downloaded the *p*-value matrix of 45,018 GO terms and 100 genes, including the gene signatures associated with SLE and breast cancer studied in the previous two sections (one gene appears in both gene lists so we have 100 genes rather than 101 genes). To simplify the data analysis, we first implemented selection of GO terms that might be more relevant to these 100 genes using the following approach. First, for each GO term, we calculated average of hypergeometric test *p*-values for the breast cancer signature genes. Then, we chose 50 GO terms with the smallest average *p*-values. Similarly, we chose 50 GO terms with the smallest average *p*-values for the 50 gene signatures associated with SLE (after excluding one gene that also appears in the breast cancer signature genes to avoid bias in the selection of GO terms due to duplicated genes) and we obtained total 100 GO terms after this step. **S3** and **S4 Tables** in the Supplementary Information show the list of GO terms selected for the gene signatures associated with SLE and breast cancer, respectively. The result indicates that the GO terms selected for the genes associated with SLE are mainly related to immune response/processes, e.g., immune response, immune cell activation/differentiation/proliferation, and interleukin activity. On the other hand, the GO terms selected for the breast cancer signature genes represent many cellular and molecular processes associated with cancer progression, such as cell cycle arrest, cell proliferation, kinase activity, and growth factor binding. Overall, these selected GO terms are considered to be well associated with the gene signatures associated with SLE and breast cancer. Finally, we took probit transformation of the resulting *p*-values for the purpose of data analyses described in detail below. We note that this transformation helps to make these values more normal-like and not bounded between 0 and 1. In addition, this transformation also shrinks background observations (*p*-values that are significantly larger than zero) around zero, while expanding signal observations (*p*-values close to zero) and pushing them away from zero, which allows to use existing statistical methods.

**Fig 4:**
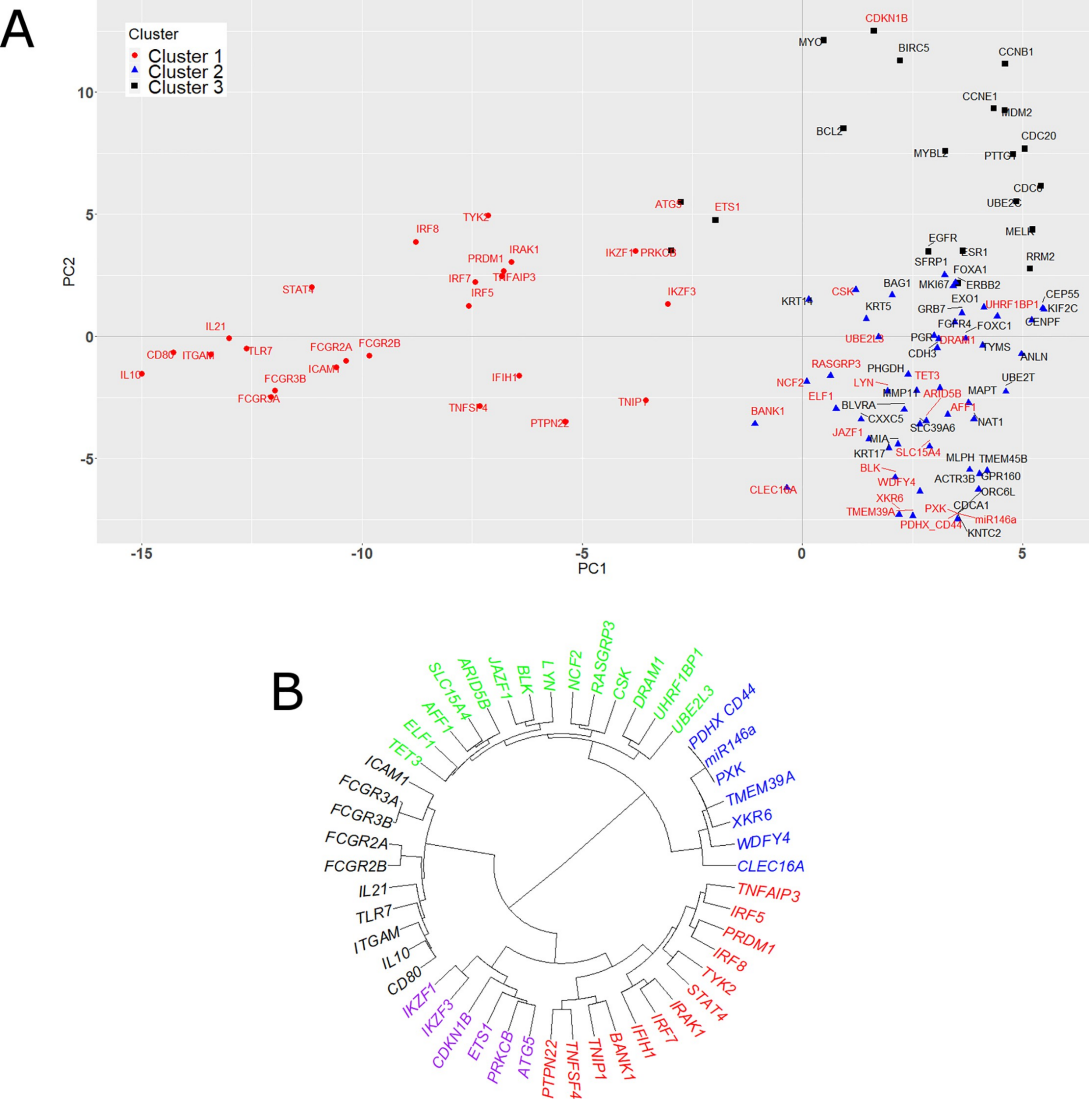
Downstream analysis using the lower-level data available in GAIL. (A) K-means clustering result for the gene signatures associated with SLE and breast cancer, using the lower-level hypergeometric test *p*-values downloaded from the GAIL web webserver. The clustering result is displayed on the first two principal components. Red circles, blue triangles, and black squares indicate cluster memberships predicted by the k-means clustering algorithm (assuming 3 clusters) while red and black texts indicate the gene signatures associated with SLE and breast cancer, respectively. (B) Dendrogram of the genes associated with SLE, constructed by applying the hierarchical clustering algorithm to the lower-level hypergeometric test *p*-values downloaded from the GAIL web webserver. Colors of gene names indicate the cluster memberships identified using the dendrogram when the number of clusters was set to five.

Using the probit-transformed hypergeometric test *p*-value matrix of 100 GO terms and 100 genes obtained from this preprocessing step, we implemented k-means clustering analysis with 3 clusters based on the visual inspection. **Fig 4A** shows the k-means clustering results, where cluster memberships are marked on the first two principal components (PC). Here, Clusters 1 (red circle) and 3 (black square) consist mostly of the gene signatures associated with SLE (red text) and breast cancer (black text), respectively, while Cluster 2 (blue triangle) has a mixed set of both SLE and breast cancer signature genes. We found that Cluster 3 contains all the communities we identified for the breast cancer signature genes using GAIL with high confidence (the cosine similarity cut-off > 0.492). Likewise, Cluster 1 contains all the communities we identified for the gene signatures associated with SLE using GAIL with high confidence. This means that the smaller PC1 scores and the larger PC1 and PC2 scores essentially indicate specific associations with SLE and breast cancer, respectively, based on the biomedical literature. On the other hand, the overlapping region (Cluster 2, which corresponds to the genes located in the bottom right corner) includes genes that are less characterized in the literature and may play as yet undefined roles in the etiology of these diseases. The heatmap of hypergeometric test *p*-values (**S2 Fig** in the Supplementary Information) confirms this finding. Specifically, the genes belonging to Clusters 1 and 3 are associated with the GO terms associated with the gene signatures associated with SLE and breast cancer, respectively. As expected, Cluster 2 genes are not associated well with the 100 GO terms in this heatmap, implying that these genes are less characterized in the literature. This conclusion still remains valid even when we considered 200 GO terms with the smallest average p-values (**S3 Fig** in the Supplementary Information).

Next, we focused on the SLE gene signatures and implemented a hierarchical clustering analysis with the Ward linkage and Euclidean distance based on probit-transformed *p*-values. **Fig 4B** shows the dendrogram, along with the clustering results when we assumed five clusters based on the visual inspection of the dendrogram. Here, the *red* cluster mostly correspond to the genes associated with the “type I interferons” and “NF-κB signalling” pathways while the *black* cluster is mainly related to the “immune complex clearance” pathway. Both of the *green* and *purple* clusters are mostly related to the “B and T cell signalling” pathway while the *blue* cluster corresponds to the genes that are not affiliated with any of these pathways. In summary, here we could nicely reveal the biology associated with SLE and breast cancer, which illustrates the potential usefulness of the lower-level data provided by GAIL for various downstream analyses. Although we presented only simple analyses in this section, more advanced statistical and computational analyses can also be implemented using this lower-level data. For example, the user can identify novel pathway-modulating genes by applying the R package ‘BayesGO’ [24] to this lower-level data provided by GAIL.

## Discussion

In this paper, we introduced GAIL (http://chunglab.io/GAIL/), our novel web interface for gene-gene association analysis based on biomedical literature mining. GAIL allows efficient inference and visualization of gene-gene relationships using community detection and enrichment analysis tools while providing more comprehensive literature mining results by utilizing GO terms. Our results for the gene signatures associated with SLE and breast cancer suggest that GAIL can provide effective means to dynamically and interactively mine relationships among genes. Currently, there are some other webservers available for text mining-based inference of gene-gene associations, including PubTator [18, 19], Literome [17], and GeneDive [20]. Compared to other available webservers, GAIL provides diverse functionalities that facilitate more immediate interpretation in the context of functional modules and systems biology, along with the data access that allows various downstream analyses (the comparison of features between GAIL and the other webservers is provided in **Table 1**). Future development directions are as follows. First, we will improve computational efficiency and performance to further scale up to a larger number of genes. Second, we will improve the user interface to further facilitate convenience of users, especially regarding the query of genes and GO terms. Third, our text mining results are currently based on co-occurrence of genes or GO terms and, ultimately, we will deploy the more improved version of GAIL based on the natural language processing (NLP) technologies to improve text mining results. In addition, we will also investigate factors that can potentially improve our text mining, such as negative words, the GO structure, publishing years of articles, and journal impacts, among others. Finally, in order to assist further investigation of users who use GAIL, we will also provide specific literature excerpts referencing the articles from which the gene-gene association is inferred from.

**Table 1:** Comparison of features between GAIL and other available webservers for text mining-based inference of gene-gene associations (GeneDive, Literome, and PubTator).

*Feature*	*GAIL*	*GeneDive*	*Literome*	*PubTator*
***Batch search***	Yes	No	No	No
***Association measure***^***a***^	Yes	Yes	No	No
***Network visualization***	Yes	Yes	No	No
***Community detection***	Yes	No	No	No
***Gene ontology analysis***	Yes	No	No	No
***Lower-level data access***	Yes	No	No	No
***Includes literature excerpts***	No	Yes	Yes	Yes
***User system***^***b***^	No	Yes	No	Yes

^a^: Whether similarity measures indicating gene-gene associations are provided.

^b^: Whether users can generate an account and keep track of their jobs.

## Materials and methods

### Text mining based on co-occurrence of genes and GO terms

**Fig 5:**
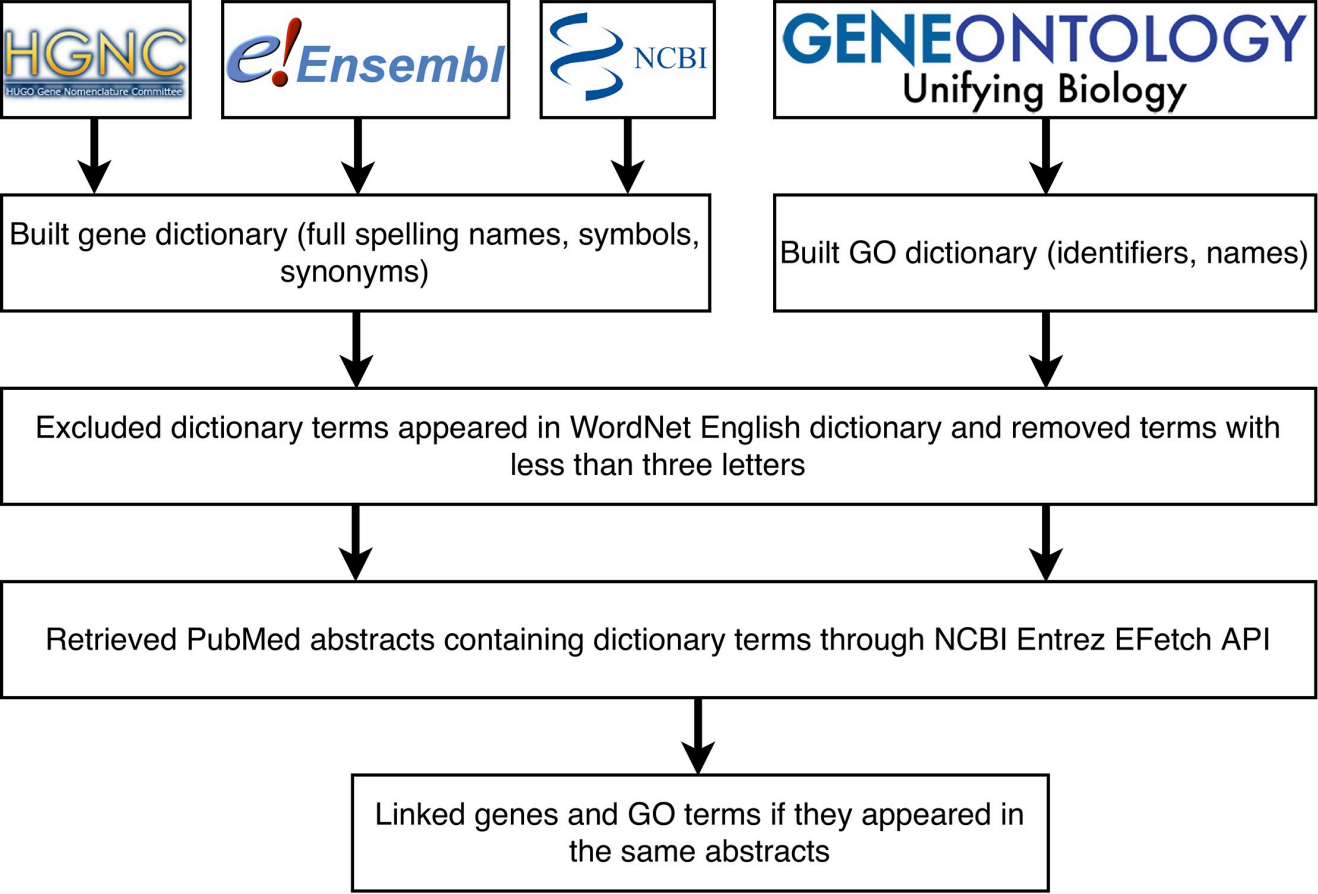
Flowchart of the term co-occurrence-based text mining approach used in GAIL. Gene names, symbols and alias were integrated from the HUGO, NCBI GenBank and Ensembl. GO names and terms were collected from the Gene Ontology Consortium.

We used the co-occurrence-based text mining framework proposed by Chung et al. [24] and Tsoi et al. [36] to extract relationships between genes. Specially, instead of searching for the co-occurrence of two gene terms, this approach searches for the co-occurrence of a gene and a GO term in the same abstract. This approach allows us to identify many indirect relationships among genes by using GO information [24, 36]. **Fig 5** summarizes our text mining process. The first step involved building pre-defined name dictionaries for genes and GO terms, respectively. Name dictionaries contained all variations of gene names and GO terms appearing in literature, including 1) full name spellings, gene symbols, and synonyms for gene names; and 2) identifiers and names for GO terms. We integrated gene names, symbols and synonyms from three databases: HGNC [37], NCBI GenBank [38], and Ensembl [39]. For GO terms, we collected their identifiers and names from the Gene Ontology Consortium [40, 41]. In total, 41,138 unique genes were collected in the gene name dictionary, and 45,000 GO terms were collected in the GO dictionary. In the second step, in order to avoid potential false positives, we removed names that correspond to common words or have less than three letters from the gene and the GO dictionaries. For instance, searching for the GO term ‘learning’ (GO: 0007612) in abstracts can return many irrelevant articles to the GO term. In order to address this, we utilized WordNet, a lexical database of general English, to filter out common words [42]. In the third step, we searched for gene and GO term occurrences in PubMed abstracts using the NCBI Entrez EFetch Application Programming Interface (API), which retrieves abstracts that match any terms in corresponding dictionaries. From this step, we obtained abstracts related to each of the genes and GO terms in the dictionaries. Finally, we linked genes and GO terms based on their co-occurrence in the same abstract.

### Gene-GO association measure

After linking genes with GO terms, we calculated a quantitative measure for association between a gene and a GO term using a hypergeometric test approach proposed earlier [24, 36]. We use subscripts *i* and *j* for genes and GO terms, respectively. We assume that the *i*-th gene occurred in *n*_*i*._ abstracts, the *j*-th GO term occurred in *n*._*j*_ abstracts, and they co-occurred in *n*_*ij*_ abstracts. The total number of abstracts mentioning at least one gene or GO term was obtained as *n*_.._ = 2,003,700. We calculated the hypergeometric test *p*-value for *i*-th gene and *j*-th GO term using the following formula:

$$p_{ij}=\sum_{n_{ij}^{\prime }>n_{ij}}^{n_{.j}}\frac{\left(\begin{array}{c} n_{.j}\\ n_{ij}^{\prime } \end{array})(\begin{array}{c} n_{..}-n_{.j}\\ n_{i.}-n_{ij}^{\prime } \end{array}\right)}{\left(\begin{array}{c} n_{..}\\ n_{i.} \end{array}\right)}+\frac{1}{2}\frac{\left(\begin{array}{c} n_{.j}\\ n_{ij} \end{array})(\begin{array}{c} n_{..}-n_{.j}\\ n_{i.}-n_{ij} \end{array}\right)}{\left(\begin{array}{c} n_{..}\\ n_{i.} \end{array}\right)},$$

where the second term was added for a continuity correction. This *p*-value measures how strong the association between a given gene and GO term is, based on the evidences from the text mining. In addition, this approach has an advantage of considering the marginal counts (*n*_*i*._ and *n*._*j*_), which reflect how much each gene and GO term has been studied in the literature.

### Gene-Gene association measure

Inference of the association between *i*-th gene and *i'*-th gene was made using the hypergeometric test *p*-values computed above. Specifically, we measured the association between *i*-th gene and *i'*-th gene by calculating a cosine similarity score:

$$\gamma_{i,i\prime }=\frac{\sum_{j=1}^{G_{ii\prime }}\left(-\log p_{ij})(-\log p_{i\prime j}\right)}{\sqrt{\sum_{j=1}^{G_{ii\prime }}{\left(-\log p_{ij}\right)}^{2}}\sqrt{\sum_{j=1}^{G_{ii\prime }}{\left(-\log p_{i\prime j}\right)}^{2}}},$$

where *G_ii_*′ is the number of GO terms associated with at least one of the *i*-th gene and the *i'*-th gene. Because cosine similarity evaluates the cosine of the angle between two vectors, a larger *γ_i,i_*′ value indicates the closer distance between the *i*-th gene and the *i'*-th gene. Note that (−log*p_ij_*) increases as *p_ij_* decreases and is bounded below at zero. Before we calculated this cosine similarity score, we truncated *p_ij_* at 10^−6^ to avoid bias due to extremely small values. If there is no hypergeometric test *p*-value for the pair of *i*-th gene and *j*-th GO term (i.e., there is no abstract mentioning this gene-GO term pair), then the corresponding (−log*p_ij_*) was set to zero. Finally, in our network construction, we consider the *i*-th gene and the *i'*-th gene to be associated if *γ_i,i_*′ ≥ *c*, where *c* is a user-determined cut-off value.

### Database set-up

To store relationships among genes, GO terms, and PubMed abstracts, we used Neo4j (https://neo4j.com/) as a backend database. Neo4j is a graph database in which data are stored using graph structures rather than table structures [43]. We prefer Neo4j over traditional databases relying on table structures because it is more efficient for storing and querying network data. In traditional databases, each relationship is commonly stored as one row in a table. In this case, whenever a gene is queried and linked GO terms are retrieved through PubMed abstracts, millions of relationships need to be searched each time. Consequently, querying relationships in the table is often computationally intensive. In contrast, Neo4j stores genes and abstracts as nodes in a graph and relationships between genes and abstracts as edges in the graph. Hence, when we query a gene in Neo4j, we only need to search the gene in all gene nodes and then traverse all edges of the gene to find GO terms connected to it, which makes querying relationships much faster in Neo4j compared to traditional databases.

### Web interface development

Using Neo4j as the backend database, we developed GAIL (http://chunglab.io/GAIL), a webserver with an interface. GAIL was built using Django, a Python web framework [44], and deployed under the Ubuntu operating system. To provide interactive and dynamic visualization of gene-gene association networks, we utilized sigma.js (http://sigmajs.org/), which is relatively lightweight and easy to integrate into web applications compared to other visualization tools [45]. We visualize a network using the Fruchterman-Reingold force-directed algorithm that positions nodes at points that minimize overlap between drawn edges, which is ideal for inspection of the gene clustering structure within the network [46]. This algorithm has a tuning parameter, *k*, the ‘optimal distance’ between nodes in the network. The value of *k* determines the space between the drawn nodes in the network: the higher value of *k* leads to the more spaced-out graph, while the lower value of *k* leads to the more tightly-drawn graph. In GAIL, users can change *c* and *k* values to modify edges and the network layout, respectively, and check the resulting network interactively to achieve the optimal network visualization.

For more rigorous investigation of the gene clustering structure, we provide a tool to detect functionally-related subgroups in a resulting gene-gene association network using the community detection algorithm. It was suggested that such detection can provide insight into functional components of a biological network and reveal groups that are highly internally connected because components in the same community often share similar functions in the biological system [47]. We used the Python package NetworkX (https://networkx.github.io/) to perform the community detection by using an asynchronous label propagation method [48]. At the start of the algorithm, each gene is first assigned a unique label. Then, in each iteration, each gene is assigned the label that appears most frequently among its connected genes. The algorithm terminates when all nodes have the labels that appear most frequently among their neighbors. In addition to the community detection functionality, we also provide a GO enrichment analysis tool to facilitate interpretation of a community’s biological function. In this enrichment analysis, the *p*-value of each GO term for the given community is computed by averaging the hypergeometric test *p*-values across all the genes belonging to the community for a given GO term. If there is no *p*-value for a given gene-GO term pair, it is considered to be one in this calculation for more conservative decision of enrichment.

### Gene lists for performance evaluation

We used two gene lists as case studies for demonstrating the utility of GAIL. The first is a list of genes associated with SLE, an autoimmune disease with poorly understood genetic factors. We used a published list of 51 genes associated with SLE [28]. This gene list contains genes that are members of five main pathways dysregulated in SLE: type I interferon signaling, NF-κB signaling, immune complex clearance, B and T cell signaling, and others. Type I interferons are involved with antimicrobial responses and also development of the adaptive and innate immune systems [49]. NF-κB signaling pathway is nearly ubiquitous in animals and is involved in many cellular responses, including the inflammatory response [50, 51]. Immune complex clearance refers to the removal of antibody-antigen complexes, and SLE has been reported to be associated with deficiencies of genes involved in this process [52, 53]. This list also contains genes that are not affiliated with any of these pathways. Of note, most of these genes have pleiotropic functions and can be placed in multiple biological pathways, but are commonly placed into broad pathways that are relevant to the disease for ease of interpretation. The second gene list contains the 50 genes in the PAM50 panel to identify breast cancer subtypes. This gene expression assay using 50 genes was developed based on global gene expression results that initially defined four breast-cancer subtypes [27]. The subtypes include Luminal A, Luminal B, HER2-enriched, and Basal-like, which are ordered by crescent aggressiveness and de-crescent cell differentiation [31, 32]. The genes were selected by how well they could separate the patients in different subtypes. Hence, these genes are not necessarily a part of the same pathway or gene family [31, 32]. The PAM50 assay has greatly improved patient diagnosis and treatment options [31, 32].

## Supporting information

S1 Fig Comparison of gene-gene networks produced by GAIL vs. a gene co-occurrence approach. The number of edges observed among the breast cancer signature genes are plotted for each method over a range of cosine similarity percentile thresholds.
(TIFF)

S2 Fig Heatmap of hypergeometric test *p*-values. The genes associated with SLE and the breast cancer signature genes (columns) and the 100 GO terms associated with these genes (rows).
(TIFF)

S3 Fig Heatmap of hypergeometric test *p*-values. The genes associated with SLE and the breast cancer signature genes (columns) and the 200 GO terms associated with these genes (rows).
(TIFF)

S1 Table List of the gene signatures associated with breast cancer. (DOCX)

S2 Table List of the gene signatures associated with SLE. (DOCX)

S3 Table List of 50 GO terms selected for the gene signatures associated with SLE. (DOCX)

S4 Table List of 50 GO terms selected for the gene signatures associated with breast cancer. (DOCX)
