# Supplementary figures and images for: GAIL: An interactive webserver for inference and dynamic visualization of gene-gene associations based on gene ontology guided mining of biomedical literature
Source: PLoS One. 2019 Jul 1;14(7):e0219195. doi: 10.1371/journal.pone.0219195 (PMC6602258; doi:10.1371/journal.pone.0219195)

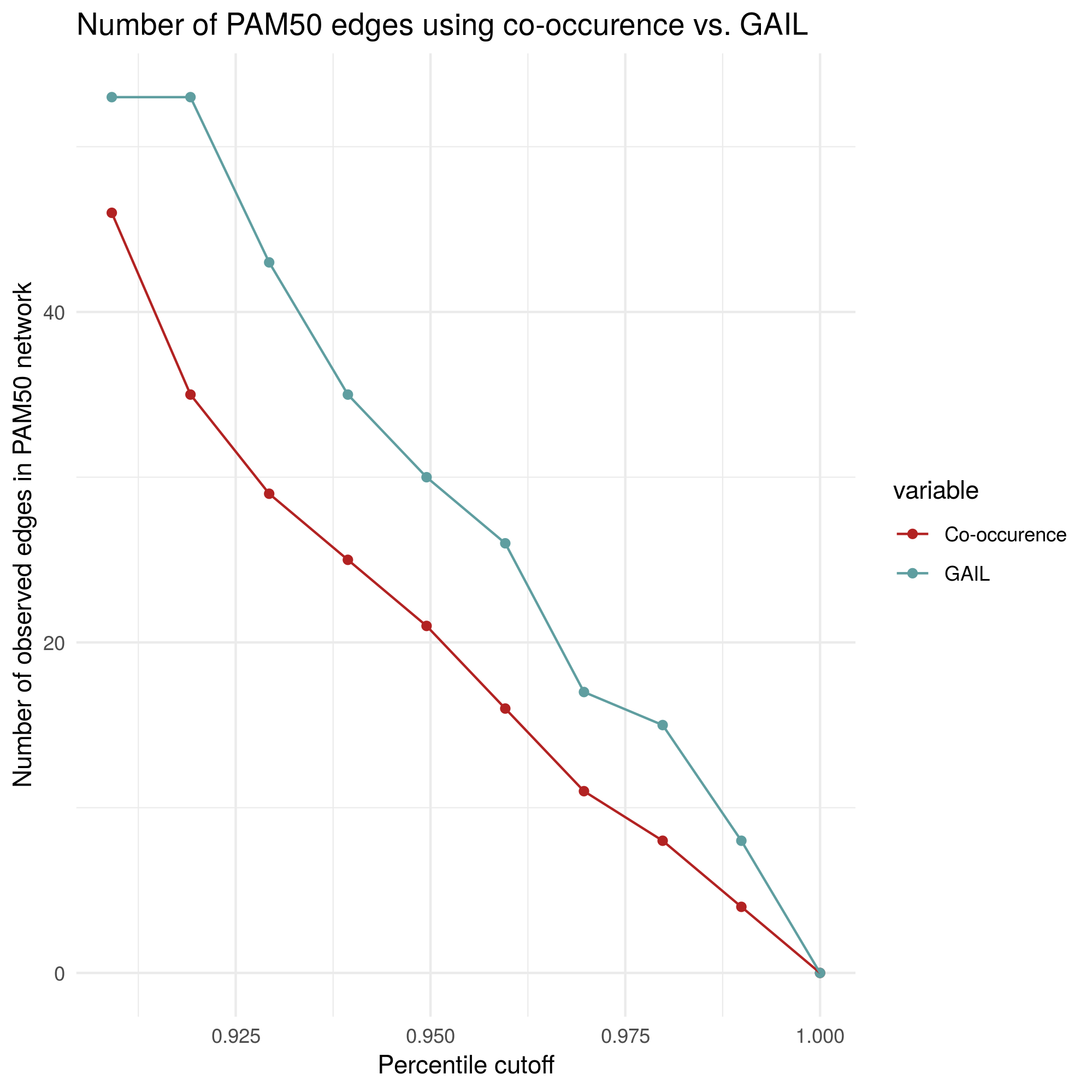

Supplement: S1 Fig — The number of edges observed among the breast cancer signature genes are plotted for each method over a range of cosine similarity percentile thresholds. (TIFF) [file pone.0219195.s001.tiff]

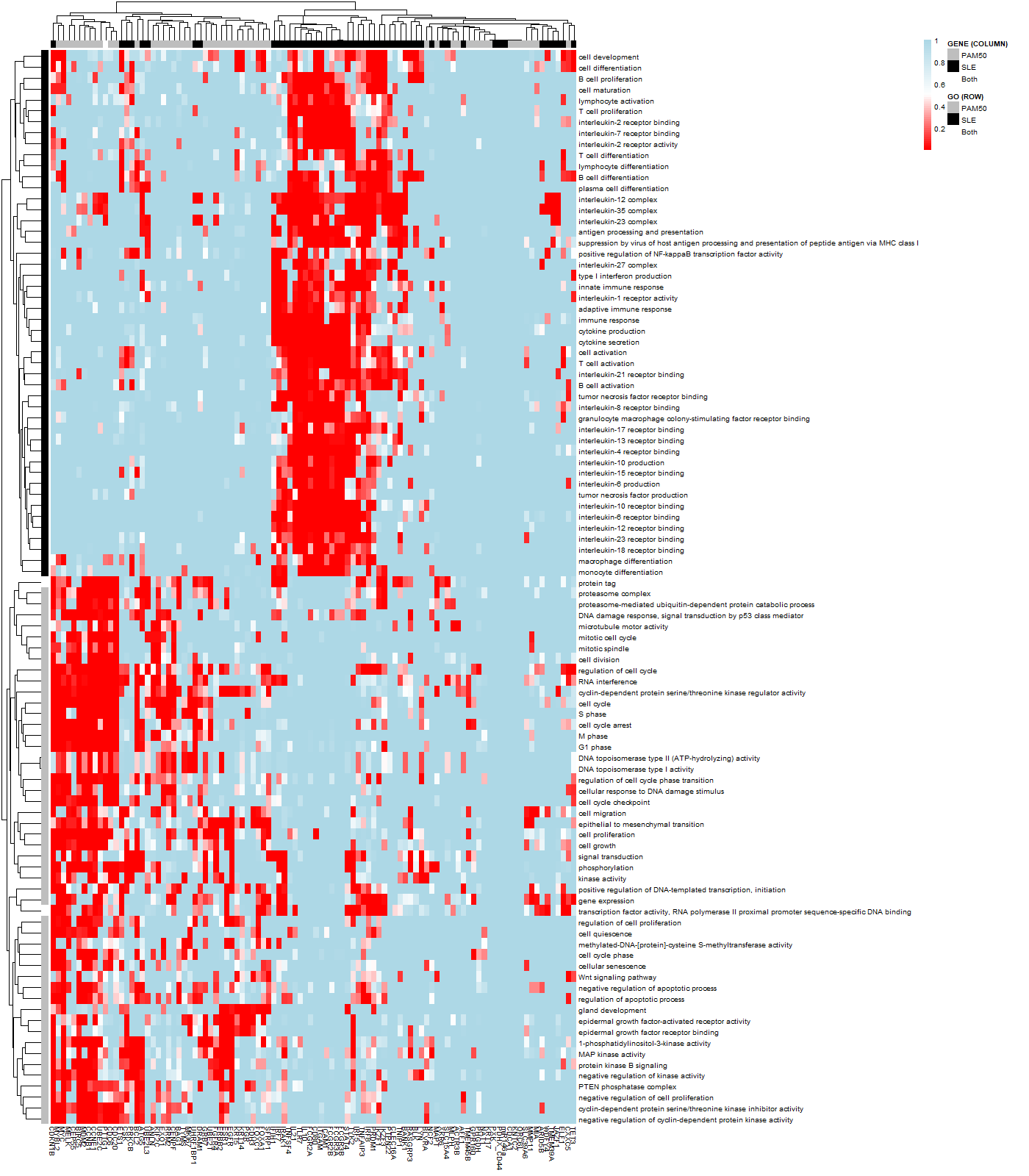

Supplement: S2 Fig — The genes associated with SLE and the breast cancer signature genes (columns) and the 100 GO terms associated with these genes (rows). (TIFF) [file pone.0219195.s002.tiff]

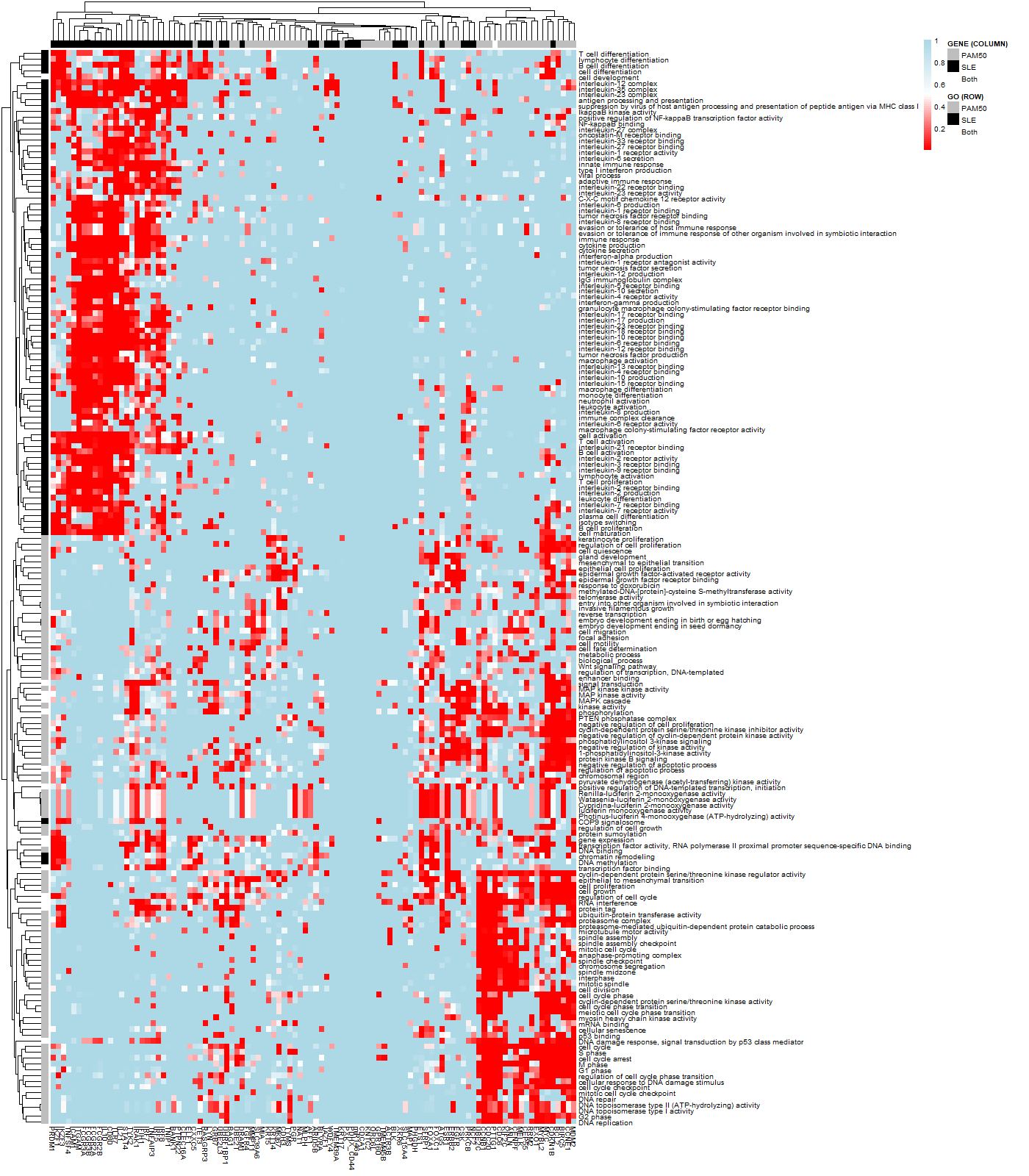

Supplement: S3 Fig — The genes associated with SLE and the breast cancer signature genes (columns) and the 200 GO terms associated with these genes (rows). (TIFF) [file pone.0219195.s003.tiff]
