# Supplementary material for: GAIL: An interactive webserver for inference and dynamic visualization of gene-gene associations based on gene ontology guided mining of biomedical literature
Source: PLoS One. 2019 Jul 1;14(7):e0219195. doi: 10.1371/journal.pone.0219195 (PMC6602258; doi:10.1371/journal.pone.0219195)
Supplement: S1 Table — (DOCX) [file pone.0219195.s004.docx]

**S1 Table**. List of the gene signatures associated with breast cancer.

| \| *UBE2C* \| \| --- \| \| *PTTG1* \| \| *MYBL2* \| \| *BIRC5* \| \| *CCNB1* \| \| *TYMS* \| \| *MELK* \| \| *CEP55* \| \| *KNTC2* \| \| *UBE2T* \| \| *RRM2* \| \| *CDC6* \| \| *ANLN* \| \| *ORC6L* \| \| *KIF2C* \| \| *EXO1* \| \| *CDCA1* \| \| *CENPF* \| \| *CCNE1* \| \| *MKI67* \| \| *CDC20* \| \| *MMP11* \| \| *GRB7* \| \| *ERBB2* \| \| *TMEM45B* \| \| *BAG1* \| \| *PGR* \| \| *MAPT* \| \| *NAT1* \| \| *GPR160* \| \| *FOXA1* \| \| *BLVRA* \| \| *CXXC5* \| \| *ESR1* \| \| *SLC39A6* \| \| *KRT17* \| \| *KRT5* \| \| *SFRP1* \| \| *BCL2* \| \| *KRT14* \| \| *MLPH* \| \| *MDM2* \| \| *FGFR4* \| \| *MYC* \| \| *MIA* \| \| *FOXC1* \| \| *ACTR3B* \| \| *PHGDH* \| \| *CDH3* \| \| *EGFR* \| |
| --- | --- | --- | --- | --- | --- | --- | --- | --- | --- | --- | --- | --- | --- | --- | --- | --- | --- | --- | --- | --- | --- | --- | --- | --- | --- | --- | --- | --- | --- | --- | --- | --- | --- | --- | --- | --- | --- | --- | --- | --- | --- | --- | --- | --- | --- | --- | --- | --- | --- | --- |
