# Supplementary material for: GAIL: An interactive webserver for inference and dynamic visualization of gene-gene associations based on gene ontology guided mining of biomedical literature
Source: PLoS One. 2019 Jul 1;14(7):e0219195. doi: 10.1371/journal.pone.0219195 (PMC6602258; doi:10.1371/journal.pone.0219195)
Supplement: S2 Table — (DOCX) [file pone.0219195.s005.docx]

**S2 Table**. List of the gene signatures associated with SLE.

| \| *IFIH1* \| \| --- \| \| *STAT4* \| \| *miR146a* \| \| *IRF5* \| \| *IRF7* \| \| *SLC15A4* \| \| *IRF8* \| \| *TYK2* \| \| *TLR7* \| \| *TNIP1* \| \| *TNFAIP3* \| \| *UBE2L3* \| \| *IRAK1* \| \| *PTPN22* \| \| *TNFSF4* \| \| *IL10* \| \| *RASGRP3* \| \| *CD80* \| \| *AFF1* \| \| *BANK1* \| \| *IL21* \| \| *PRDM1* \| \| *IKZF1* \| \| *BLK* \| \| *LYN* \| \| *ARID5B* \| \| *PDHX_CD44* \| \| *ETS1* \| \| *ELF1* \| \| *CSK*  *PTTG1* \| \| *PRKCB* \| \| *IKZF3* \| \| *ICAM1* \| \| *FCGR2A* \| \| *FCGR3A* \| \| *FCGR2B* \| \| *FCGR3B* \| \| *ITGAM* \| \| *NCF2* \| \| *TET3* \| \| *PXK* \| \| *TMEM39A* \| \| *UHRF1BP1* \| \| *ATG5* \| \| *JAZF1* \| \| *XKR6* \| \| *WDFY4* \| \| *CDKN1B* \| \| *DRAM1* \| \| *CLEC16A* \| |
| --- | --- | --- | --- | --- | --- | --- | --- | --- | --- | --- | --- | --- | --- | --- | --- | --- | --- | --- | --- | --- | --- | --- | --- | --- | --- | --- | --- | --- | --- | --- | --- | --- | --- | --- | --- | --- | --- | --- | --- | --- | --- | --- | --- | --- | --- | --- | --- | --- | --- | --- |
