# Supplementary material for: GAIL: An interactive webserver for inference and dynamic visualization of gene-gene associations based on gene ontology guided mining of biomedical literature
Source: PLoS One. 2019 Jul 1;14(7):e0219195. doi: 10.1371/journal.pone.0219195 (PMC6602258; doi:10.1371/journal.pone.0219195)
Supplement: S3 Table — (DOCX) [file pone.0219195.s006.docx]

**S3 Table**: List of 50 GO terms selected for the gene signatures associated with SLE.

| GO ID | GO term description |
| --- | --- |
| GO:0043514 | interleukin-12 complex |
| GO:0001775 | cell activation |
| GO:0070745 | interleukin-35 complex |
| GO:0030183 | B cell differentiation |
| GO:0070743 | interleukin-23 complex |
| GO:0048468 | cell development |
| GO:0042110 | T cell activation |
| GO:0001531 | interleukin-21 receptor binding |
| GO:0045087 | innate immune response |
| GO:0042113 | B cell activation |
| GO:0019882 | antigen processing and presentation |
| GO:0046776 | suppression by virus of host antigen processing and presentation of peptide antigen via MHC class I |
| GO:0001816 | cytokine production |
| GO:0042100 | B cell proliferation |
| GO:0048469 | cell maturation |
| GO:0030154 | cell differentiation |
| GO:0002250 | adaptive immune response |
| GO:0006955 | immune response |
| GO:0030217 | T cell differentiation |
| GO:0032606 | type I interferon production |
| GO:0046649 | lymphocyte activation |
| GO:0005141 | interleukin-10 receptor binding |
| GO:0042098 | T cell proliferation |
| GO:0005138 | interleukin-6 receptor binding |
| GO:0045519 | interleukin-23 receptor binding |
| GO:0004908 | interleukin-1 receptor activity |
| GO:0030367 | interleukin-17 receptor binding |
| GO:0005164 | tumor necrosis factor receptor binding |
| GO:0050663 | cytokine secretion |
| GO:0032613 | interleukin-10 production |
| GO:0000982 | transcription factor activity, RNA polymerase II proximal promoter sequence-specific DNA binding |
| GO:0051092 | positive regulation of NF-kappaB transcription factor activity |
| GO:0031386 | protein tag |
| GO:0002317 | plasma cell differentiation |
| GO:0005129 | granulocyte macrophage colony-stimulating factor receptor binding |
| GO:0005144 | interleukin-13 receptor binding |
| GO:0030225 | macrophage differentiation |
| GO:0005143 | interleukin-12 receptor binding |
| GO:0030224 | monocyte differentiation |
| GO:0016170 | interleukin-15 receptor binding |
| GO:0005136 | interleukin-4 receptor binding |
| GO:0005139 | interleukin-7 receptor binding |
| GO:0005153 | interleukin-8 receptor binding |
| GO:0045515 | interleukin-18 receptor binding |
| GO:0032635 | interleukin-6 production |
| GO:0030098 | lymphocyte differentiation |
| GO:0032640 | tumor necrosis factor production |
| GO:0070744 | interleukin-27 complex |
| GO:0005134 | interleukin-2 receptor binding |
| GO:0004911 | interleukin-2 receptor activity |
