# Supplementary material for: GAIL: An interactive webserver for inference and dynamic visualization of gene-gene associations based on gene ontology guided mining of biomedical literature
Source: PLoS One. 2019 Jul 1;14(7):e0219195. doi: 10.1371/journal.pone.0219195 (PMC6602258; doi:10.1371/journal.pone.0219195)
Supplement: S4 Table — (DOCX) [file pone.0219195.s007.docx]

**S4 Table**. List of 50 GO terms selected for the gene signatures associated with breast cancer.

| GO ID | GO term description |
| --- | --- |
| GO:0016538 | cyclin-dependent protein serine/threonine kinase regulator activity |
| GO:0051726 | regulation of cell cycle |
| GO:0016246 | RNA interference |
| GO:0008283 | cell proliferation |
| GO:0001837 | epithelial to mesenchymal transition |
| GO:0007049 | cell cycle |
| GO:0007050 | cell cycle arrest |
| GO:0016049 | cell growth |
| GO:0004861 | cyclin-dependent protein serine/threonine kinase inhibitor activity |
| GO:0000279 | M phase |
| GO:0010467 | gene expression |
| GO:1901987 | regulation of cell cycle phase transition |
| GO:0016477 | cell migration |
| GO:0051320 | S phase |
| GO:0005006 | epidermal growth factor-activated receptor activity |
| GO:0016303 | 1-phosphatidylinositol-3-kinase activity |
| GO:0000502 | proteasome complex |
| GO:0016310 | phosphorylation |
| GO:0051318 | G1 phase |
| GO:0022403 | cell cycle phase |
| GO:0003918 | DNA topoisomerase type II (ATP-hydrolyzing) activity |
| GO:0003917 | DNA topoisomerase type I activity |
| GO:1990455 | PTEN phosphatase complex |
| GO:0043491 | protein kinase B signaling |
| GO:0043066 | negative regulation of apoptotic process |
| GO:0000982 | transcription factor activity, RNA polymerase II proximal promoter sequence-specific DNA binding |
| GO:0006974 | cellular response to DNA damage stimulus |
| GO:0000075 | cell cycle checkpoint |
| GO:1904030 | negative regulation of cyclin-dependent protein kinase activity |
| GO:2000144 | positive regulation of DNA-templated transcription, initiation |
| GO:0031386 | protein tag |
| GO:0042127 | regulation of cell proliferation |
| GO:0042981 | regulation of apoptotic process |
| GO:0043161 | proteasome-mediated ubiquitin-dependent protein catabolic process |
| GO:0004707 | MAP kinase activity |
| GO:0008285 | negative regulation of cell proliferation |
| GO:0000278 | mitotic cell cycle |
| GO:0016301 | kinase activity |
| GO:0003777 | microtubule motor activity |
| GO:0030330 | DNA damage response, signal transduction by p53 class mediator |
| GO:0090398 | cellular senescence |
| GO:0033673 | negative regulation of kinase activity |
| GO:0072686 | mitotic spindle |
| GO:0044838 | cell quiescence |
| GO:0005154 | epidermal growth factor receptor binding |
| GO:0007165 | signal transduction |
| GO:0051301 | cell division |
| GO:0003908 | methylated-DNA-[protein]-cysteine S-methyltransferase activity |
| GO:0016055 | Wnt signaling pathway |
| GO:0048732 | gland development |
